# Cold Atmospheric Plasma Does Not Affect Stellate Cells Phenotype in Pancreatic Cancer Tissue in Ovo

**DOI:** 10.3390/ijms23041954

**Published:** 2022-02-10

**Authors:** Angela Privat-Maldonado, Ruben Verloy, Edgar Cardenas Delahoz, Abraham Lin, Steve Vanlanduit, Evelien Smits, Annemie Bogaerts

**Affiliations:** 1 PLASMANT, Chemistry Department, Faculty of Sciences, University of Antwerp, 2610 Antwerp, Belgium; ruben.verloy@uantwerpen.be (R.V.); abraham.lin@uantwerpen.be (A.L.); annemie.bogaerts@uantwerpen.be (A.B.); 2 Solid Tumor Immunology Group, Center for Oncological Research, Integrated Personalized and Precision Oncology Network, Department of Molecular Imaging, Pathology, Radiotherapy and Oncology, University of Antwerp, 2610 Antwerp, Belgium; evelien.smits@uantwerpen.be; 3 Industrial Vision Lab InViLab, Faculty of Applied Engineering, University of Antwerp, 2610 Antwerp, Belgium; edgar.cardenas@uantwerpen.be (E.C.D.); steve.vanlanduit@uantwerpen.be (S.V.)

**Keywords:** PDAC, oxidative stress, pancreatic stellate cells, cold atmospheric plasma, in ovo tissue, reactive species

## Abstract

Pancreatic ductal adenocarcinoma (PDAC) is a challenging neoplastic disease, mainly due to the development of resistance to radio- and chemotherapy. Cold atmospheric plasma (CAP) is an alternative technology that can eliminate cancer cells through oxidative damage, as shown in vitro, in ovo, and in vivo. However, how CAP affects the pancreatic stellate cells (PSCs), key players in the invasion and metastasis of PDAC, is poorly understood. This study aims to determine the effect of an anti-PDAC CAP treatment on PSCs tissue developed in ovo using mono- and co-cultures of RLT-PSC (PSCs) and Mia PaCa-2 cells (PDAC). We measured tissue reduction upon CAP treatment and mRNA expression of PSC activation markers and extracellular matrix (ECM) remodelling factors via qRT-PCR. Protein expression of selected markers was confirmed via immunohistochemistry. CAP inhibited growth in Mia PaCa-2 and co-cultured tissue, but its effectiveness was reduced in the latter, which correlates with reduced ki67 levels. CAP did not alter the mRNA expression of PSC activation and ECM remodelling markers. No changes in MMP2 and MMP9 expression were observed in RLT-PSCs, but small changes were observed in Mia PaCa-2 cells. Our findings support the ability of CAP to eliminate PDAC cells, without altering the PSCs.

## 1. Introduction

Pancreatic ductal adenocarcinoma (PDAC) is the most prevalent oncologic disease of the pancreas, responsible for approximately 85% of all pancreatic malignancies [1]. Even when significant efforts have been made to improve the detection and treatment of PDAC, current therapies can only provide a five-year survival rate of up to 10% [2], which highlights the need to develop better strategies. A limiting factor of the main current therapies is that most of them target only the cancer cells, without considering the key role played by stromal cells. PDAC is characterized by its fast progression and desmoplastic reaction, orchestrated by the interplay between cancer cells and the surrounding tumour stromal cells [3]. The thick desmoplastic stroma accounts for up to 90% of the tumour volume and contains endothelial cells, immune cells, collapsed blood vessels, as well as pancreatic stellate cells (PSCs), which deposit large amounts of extracellular matrix components [4]. The dense stroma in PDAC facilitates the development of a hypoxic environment and creates a physical barrier that increases the resistance to radio- and chemotherapy [5,6]. PSCs make up 4–7% of pancreatic cells in normal tissue and are present in a quiescent state, where they regulate the production and turnover of extracellular matrix (ECM) components [6]. However, when PSCs become active (due to pancreatic injury, inflammation, oxidative stress, etc.), they acquire a myofibroblast-like phenotype characterized by the expression of α-smooth muscle actin (ACTA-2) [4]. Activated PSCs synthesize excessive amounts of ECM proteins, matrix metalloproteinases (MMP) and their inhibitors. In addition, PSCs secrete growth factors and cytokines that exert paracrine and autocrine effects on cells, conferring more proliferative and migratory abilities [6]. The crosstalk between PSCs and PDAC cells creates a specific tumour microenvironment (TME) that accelerates the proliferation of PDAC cells, inhibits their apoptosis and can induce their epithelial-mesenchymal transition (EMT), making them more migratory [7].

Reactive oxygen species (ROS) can initiate the transformation of normal to malignant cells, and the increased ROS production is key for PDAC progression. Cancer cells that adapt to the new redox state can avoid apoptosis and modulate the antioxidant systems [8]. In addition, ROS can activate the PSCs, which facilitate the infiltration of immune, endothelial, and neuronal cells into the TME [9]. However, the increased production of ROS in cancer cells put them under higher oxidative stress compared to normal cells, and a further increase in ROS levels can lead to cell death [10]. Recent studies have shown that pro-oxidant compounds can activate the protective antioxidant response in healthy cells while killing cancer cells, as the excess of ROS can quickly exhaust the antioxidant response available [10,11,12,13]. Even more, it has been suggested that increased levels of ROS could sensitize cancer cells to chemotherapeutic drugs and radiotherapy [13,14]. This highlights the potential clinical application of pro-oxidant therapies.

Therefore, novel therapies that rely on the production and delivery of reactive species, such as cold atmospheric plasma (CAP), arise as an interesting cancer treatment. CAP is a partially ionized gas made of physical and chemical components. It is well accepted that the main factors responsible for the anticancer properties of CAP are the reactive oxygen and nitrogen species (RONS), which include hydrogen peroxide (H_2_O_2_), ozone (O_3_), hydroxyl radicals (OH), superoxide (O_2_^−^), singlet oxygen (^1^O_2_), nitric oxide (NO), nitrite (NO_2_^−^), peroxynitrite (ONOO^−^), among others [15,16]. Other physical components (UV radiation, electric fields, visible light, etc.) have been shown to have a minor direct effect on biological samples and rather contribute to the formation of chemical species [17]. CAP eliminates cancer cells via apoptosis, necrosis, ferroptosis, and immunogenic cell death, as shown in in vitro, in ovo, and in vivo models [16,18,19,20], with less damaging effects on healthy cells [9,21]. The effect of CAP is not limited to only the cancer cells, as CAP can also oxidize ECM components, such as hyaluronan [22], and improve drug delivery in cancer [23,24,25]. In low doses, however, CAP-generated RONS can have positive effects, as CAP can boost wound healing and decontamination [26]. One of the most studied CAP devices for biomedical application is the kINPen [15], which has been proved to effectively eliminate PDAC cells in vitro (two- and three-dimensional models) and in ovo [27,28].

As pro-oxidant therapies could enhance the elimination of PDAC cells, it is important to consider how CAP-generated RONS affect the PSCs in PDAC. It has been shown that the addition of 50 μM H_2_O_2_ promoted the expression of ACTA-2 and induced the migration of PSCs in vitro [29]. A previous study has shown that CAP reduced PDAC cell migration in a three-dimensional cell-matrix model, without altering the migratory ability of PSCs [27]. In addition, CAP-treated solutions have been shown to be cytotoxic to PSCs, which could reduce the number of activated PSCs secreting ECM components that build up the desmoplastic barrier [30,31]. Yet, there is limited information on how CAP affects the PSC in the complex TME of PDAC, as these cells control the remodelling of the tumour architecture and tumour progression [32].

The aim of this study is to determine the effect of an anti-PDAC CAP treatment on PSCs, using a vascularized in ovo model. For this, we developed human tissue in the chicken chorioallantoic membrane (CAM) model, using mono- and co-cultures of RLT-PSC (stellate cells) and Mia PaCa-2 (PDAC cells), and delivered CAP treatments with the kINPen. We assessed the expression of markers of PSC activation and ECM remodelling factors. Our data indicate that CAP does not have a negative effect on the PSCs, while eliminating PDAC cells in a complex system.

## 2. Results

### 2.1. CAP Inhibits Growth of Mia PaCa-2 Tissue, but Effectiveness Is Reduced in the Presence of RLT-PSCs

**Figure 1:**
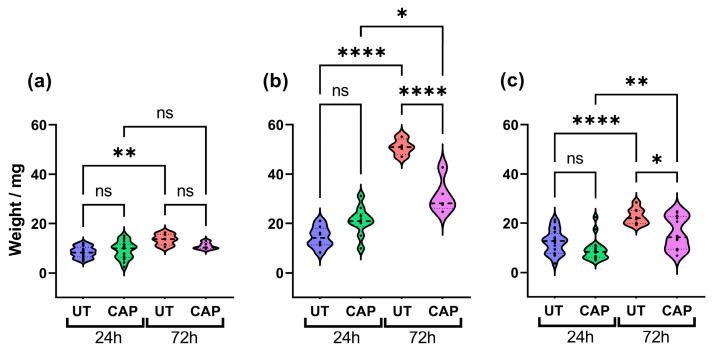
CAP inhibits tissue growth. Effect of CAP on tissue growth of (**a**) RLT-PSC, (**b**) Mia PaCa-2, and (**c**) RLT-PSC + Mia PaCa-2. CAP hinders the growth of co-cultured tissue, decreasing its size 72 h post treatment. *n* ≥ 4 tissue per condition. UT = untreated control. Each dot represents one tissue. One-way ANOVA with post-hoc Tukey’s test. Dashed lines = median; dotted lines = 25% and 75% quartiles; * = *p* ≤ 0.05; ** = *p* ≤ 0.01; **** = *p* ≤ 0.0001; ns = not significant.

To determine if CAP can reduce the size of human tissue in the CAM model, these were excised 24 h and 72 h after treatment and weighed. The results show that CAP did not alter the weight of RLT-PSC tissue (Figure 1a), which could suggest that CAP had little effect on the proliferation of these cells up to 72 h post treatment (p.t.). In contrast, although there was no significant difference between CAP-treated and untreated samples after 24 h, a significant reduction in weight was observed 72 h p.t. in CAP-treated tissue (**** = *p* ≤ 0.0001; Figure 1b). A similar trend was observed in co-cultured tissue. While untreated tissue increased in weight over time, this was hindered in CAP-treated tissue 72 h p.t. (* = *p* ≤ 0.05; Figure 1c). It is worth noting that CAP was not able to completely eliminate Mia PaCa-2 and co-cultured tissue, and CAP-treated tissue increased in weight after 72 h. However, CAP-treated tissue weighed significantly less than untreated.

**Figure 2:**
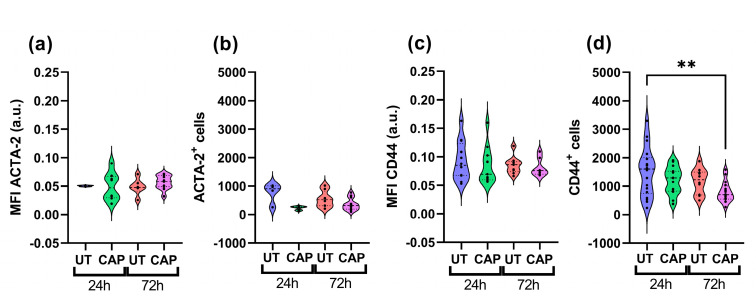
CAP reduces the population of Mia PaCa-2 cells in co-cultured in ovo tissue. Sections were stained for (**a**,**b**) ACTA-2 (RLT-PSC) and (**c**,**d**) CD44 (Mia PaCa-2). (**a**,**c**) Mean fluorescence intensity (MFI) expressed as arbitrary units (a.u.); number of cells positive for (**b**) ACTA-2 or (**d**) CD44. UT = untreated control. *n* ≥ 4 tissue per condition. Each dot represents one tissue. Dashed lines = median; dotted lines = 25% and 75% quartiles; ** = *p* ≤ 0.01.

To determine if the weight reduction observed in co-cultured tissue was due to the elimination of Mia PaCa-2 cells, we performed immunofluorescence staining with specific markers for each population. Co-cultured tissue was stained for ACTA-2, an activation marker expressed in RLT-PSC [33], and CD44, a stem cell marker of PDAC highly expressed in Mia PaCa-2 [34]. We observed that the mean fluorescence intensity (MFI) of ACTA-2 and total number of ACTA-2^+^ cells did not vary with CAP treatments, suggesting little effect of CAP on RLT-PSC (Figure 2a,b). In contrast, Mia PaCa-2 cells showed a decrease in the expression of CD44, and it corresponded to a reduced number of CD44^+^ cells 72 h p.t. (Figure 2c,d). This reduction after 72 h was significant when compared to the UT group at 24 h (** = *p* ≤ 0.01) and less so when compared with the UT group at 72 h (*p* ≥ 0.05).

The reduction in tissue weight could be due to the partial elimination of Mia PaCa-2 cells by CAP, as there is a modest trend towards the decrease of CD44^+^ cells in co-cultured tissue 72 h p.t.

### 2.2. CAP Decreases Ki67 Expression in RLT-PSC + Mia PaCa-2 Tissue

To further characterize the inhibitory ability of CAP, we evaluated the expression of the proliferation marker ki67 via immunohistochemistry. The results were scored using a semi-automated analysis with QuPath (see Materials and Methods), and the results were classified as weak (1+), moderate (2+), or strong (3+). The scoring represents the intensity of the signal found for each cell, 1+ being cells with low expression of ki67 and 3+ cells with high expression of ki67.

**Figure 3:**
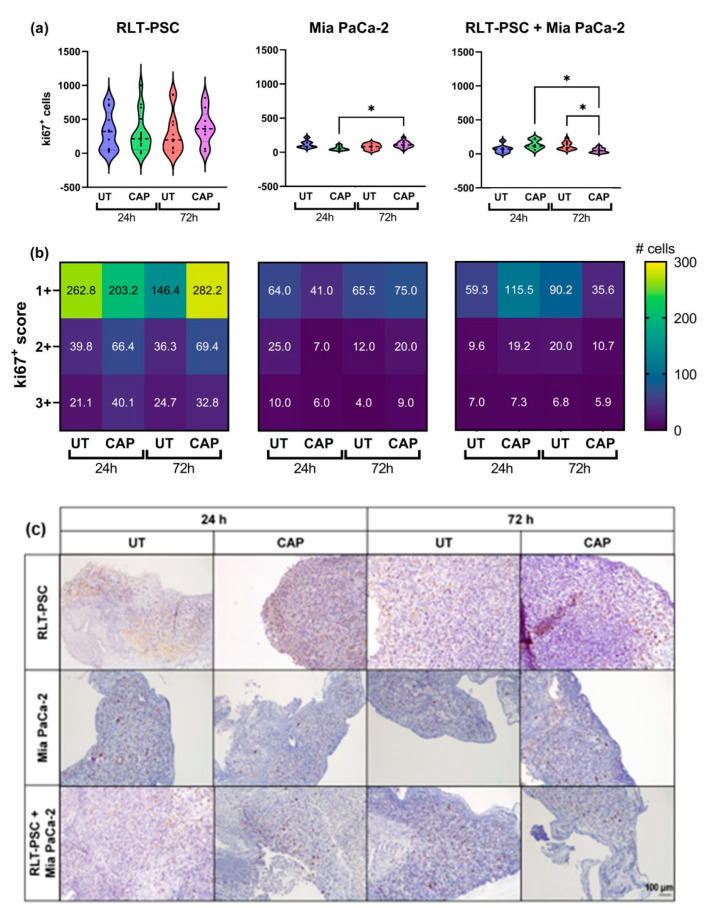
Ki67 expression in RLT-PSC, Mia PaCa-2, and RLT-PSC + Mia PaCa-2 tissue. (**a**) Total ki67^+^ counts for each tissue per treatment condition. Mixed-effects model (REML) with post-hoc Tukey’s test. Each dot represents one tissue. Dashed lines = median; dotted lines = 25% and 75% quartiles; * = *p* ≤ 0.05. (**b**) Tissue sections stained for Ki67 were scored using QuPath. Positive cells were classified as weak (1+), moderate (2+), or strong (3+) DAB signal. Heat maps present the mean values. UT = untreated control. *n* ≥ 4 tissue per condition. (**c**) Representative original images of samples stained for ki67 (DAB) and counterstained with haematoxylin (10× objective). Scale bar = 100 µm.

In general, in ovo tissue of Mia PaCa-2 only and co-cultures presented a lower number of ki67^+^ cells than RLT-PSC only tissue (Figure 3). Although CAP did not increase the weight of RLT-PSC tissue nor the total number of ki67^+^ cells (Figure 3a), there was a small increase in the number of ki67^+^ cells with a score of 1+ (weak). Surprisingly, CAP induced an increase in the number of ki67^+^ cells in Mia PaCa-2 tissue (Figure 3a,b), which could explain the tissue growth observed in Figure 1b. However, this proliferative state did not increase the tissue weight beyond the values obtained in UT tissue 72 h p.t., and the weight of CAP-treated Mia PaCa-2 tissue remained lower that the UT controls.

Importantly, CAP-treated co-cultured in ovo tissue showed a significant reduction in the number of ki67^+^ cells 72 h p.t. (* = *p* ≤ 0.05; Figure 3a) in all three scores, but most significantly in cells with a 1+ score (Figure 3b). This suggests that the co-culture of RLT-PSC and Mia PaCa-2 cells in the in ovo model downregulates the cell proliferation of stellate cells, and CAP diminishes the overall proliferative state of the tissue.

Overall, these results suggest CAP reduces the ki67 expression in co-cultured tissue of RLT-PSC and Mia PaCa-2 cells.

### 2.3. CAP Does Not Alter mRNA Expression of Activation, Cell-Matrix Interaction, and ECM Remodelling Factors in Tissue with RLT-PSC Cells

**Figure 4:**
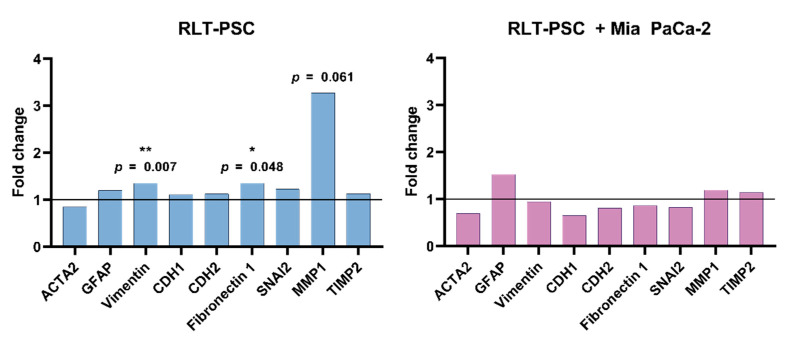
CAP does not alter mRNA expression of activation and ECM remodelling factors in tissue with RLT-PSC cells. mRNA was extracted from RLT-PSC and RLT-PSC + Mia PaCa-2 tissue. Data are presented in arbitrary units of the calculated relative normalized expression based on the untreated controls (ΔΔCq; fold change 1 = untreated controls). Analysis done with the Bio-Rad CFX Maestro 1.1 software.

To determine if CAP could alter the expression of markers related to activation of stellate cells (ACTA-2, GFAP, vimentin), cell-matrix interaction (CDH1, CDH2, fibronectin 1, SNAI2), and ECM remodelling (MMP1, TIMP2), we performed qRT-PCR of single RLT-PSC and co-cultured tissue 24 h p.t. We observed that CAP did not affect the activation profile of RLT-PSC and co-cultured tissue (Figure 4), as there was no variation in the mRNA levels of both UT and CAP-treated samples. Although vimentin and fibronectin 1 were significantly altered (*p* ≤ 0.05), the fold change was small. The slight increase in MMP1 expression in RLT-PSC was not statistically significant (*p* = 0.061), even when the fold change was over 2.

Previous characterization of the RLT-PSC cells has shown that these cells present an activated phenotype [35]. Thus, we conclude that CAP does not alter the mRNA expression of activation markers of RLT-PSC cells, nor the expression of the ECM remodelling and cell-matrix interaction factors studied here.

### 2.4. CAP Does Not Alter the Activation Profile of RLT-PSC Cells in Ovo

**Figure 5:**
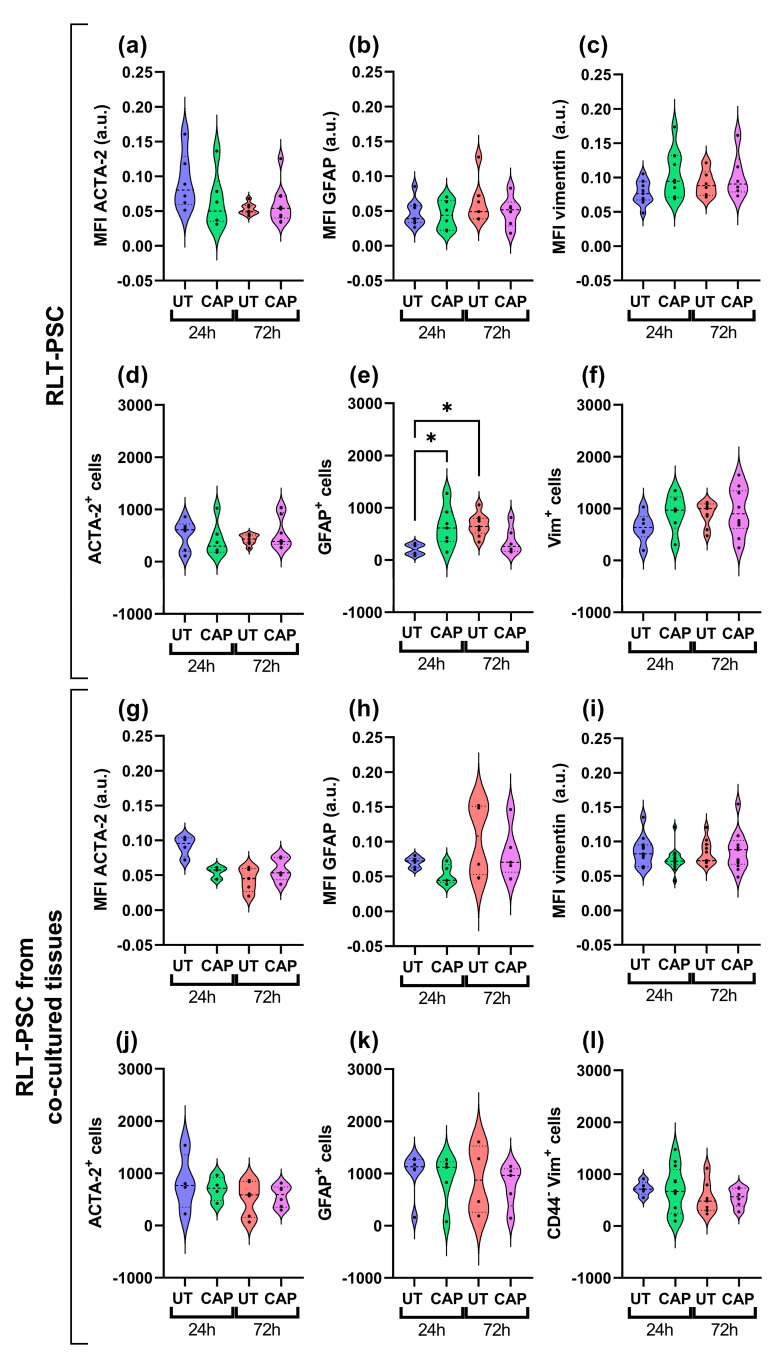
CAP does not alter the activation profile of RLT-PSC cells in ovo. Sections of RLT-PSC and co-cultured RLT-PSC + Mia PaCa-2 from in ovo tissue were stained for GFAP, vimentin (Vim), and ACTA-2. (**a**–**c**,**g**–**i**) Mean fluorescence intensity (MFI) expressed as arbitrary units (a.u.); (**d**–**f**,**j**–**l**) number of cells positive for the corresponding markers. Anti-CD44 antibodies (PDAC cells) were used as a counterstain to discriminate Mia PaCa-2 cells from RLT-PSC. UT = untreated control. *n* ≥ 4 tissue per condition; each dot represents one tissue. Dashed lines = median; dotted lines = 25% and 75% quartiles; * = *p* ≤ 0.05.

Immunofluorescence staining was performed from resected tissue. The paraffin sections revealed that, overall, there was no variation in the expression of activation markers GFAP, vimentin, and ACTA-2 in RLT-PSCs. This was observed in both single RLT-PSC tissue (Figure 5a–c) and co-cultured tissue (Figure 5g–i). Accordingly, the number of cells expressing these markers remained unchanged in CAP-treated tissues after 24 and 72 h (Figure 5d–f,j–l). We identified an increase in the number of cells expressing GFAP in CAP-treated RLT-PSC tissue 24 h p.t. compared to the untreated samples, however, GFAP expression returned to baseline after 72 h.

Our results suggest that CAP treatment does not alter the activation state of RLT-PSC cells when alone or in co-culture with PDAC cells in the in ovo model.

### 2.5. CAP Does Not Affect MMPs Expression in RLT-PSC but Increases MMP2 Expression in Mia PaCa-2 of Co-Cultured Tissue

**Figure 6:**
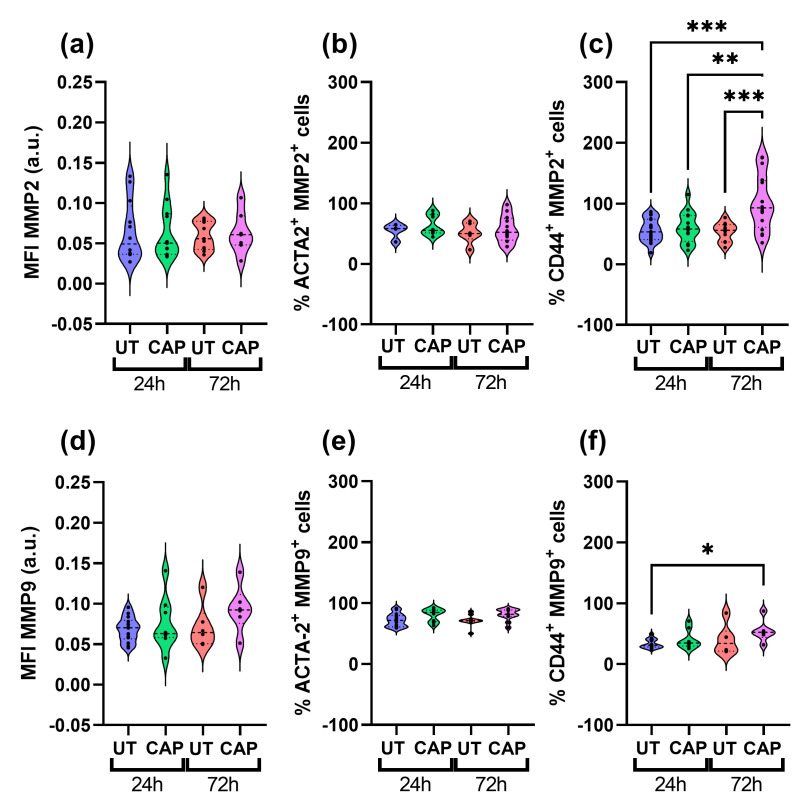
MMPs expression in co-cultured RLT-PSC + Mia PaCa-2 in ovo tissue. Tissue sections were stained for MMP2 and MMP9 in co-cultured tissue, using CD44 and ACTA-2 as specific markers for Mia PaCa-2 and RLT-PSC, respectively. (**a**,**d**) Mean fluorescence intensity (MFI) expressed as arbitrary units (a.u.). Percentage of cells positive for (**b**) ACTA-2 and MMP2, (**c**) CD44 and MMP2, (**e**) ACTA-2 and MMP9, and (**f**) CD44 and MMP9. UT = untreated control. *n* ≥ 4 tissue per condition. Each dot represents one tissue. Dashed lines = median; dotted lines = 25% and 75% quartiles; * = *p* ≤ 0.05; ** = *p* ≤ 0.01; *** = *p* ≤ 0.001.

We also measured the expression of MMP2 and MMP9 in both populations of cells present in the in ovo tissue. Both proteins are type IV collagenases, known to degrade major structural proteins of the ECM and basement membrane, thus actively participating in ECM remodelling and EMT [36,37]. CAP-treated samples had similar MFI values for MMP2 and MMP9 compared to UT samples (Figure 6a,d), as there was no statistically significant difference between the UT and CAP groups 72 h p.t.

When looking at the percentage of ACTA-2^+^ cells that also express MMP2 and MMP9, it is clear that CAP does not affect their expression in RLT-PSC cells (Figure 6b,e). However, we observed a significant increase in the percentage of CD44^+^ MMP2^+^ cells in co-cultured tissue after 72 h, compared to UT samples at 24 h and 72 h (*** = *p* ≤ 0.001; Figure 6c). Similarly, we observed an increase in the percentage of CD44^+^ MMP9^+^ cells over time (* = *p* ≤ 0.05; Figure 6f). The difference in MMP9 expression was only observed between the UT samples at 24 h p.t. and CAP samples at 72 h p.t., as both groups at 72 h presented similar values.

**Figure 7:**
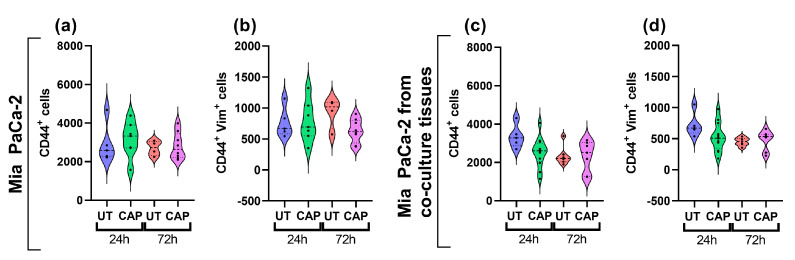
Vimentin expression in Mia PaCa-2 of single and co-cultured in ovo tissue. Tissue sections were stained for CD44 and vimentin. Number of cells positive for (**a**,**c**) CD44, (**b**,**d**) CD44 and vimentin. UT = untreated control. *n* ≥ 4 tissue per condition. Each dot represents one tissue. Dashed lines = median; dotted lines = 25% and 75% quartiles.

In addition, we assessed the expression of vimentin in Mia PaCa-2 cells of single and co-cultured tissue, as this protein is upregulated in cells undergoing EMT to increase their migratory capacity [38]. CAP did not boost the expression of vimentin in Mia PaCa-2 cells in single or co-cultured tissue, as no increase in the number of cells expressing the marker was observed (Figure 7a–d). This suggests that CAP does not alter the expression of this protein up to 72 h p.t.

Thus, we conclude that CAP does not affect the expression of MMP2 and MMP9 in RLT-PSC cells in co-cultured in ovo tissue. In Mia PaCa-2 cells from co-cultured tissue, CAP activates the expression of MMP2 and MMP9 but not vimentin, which suggests CAP might indirectly contribute to the modification of the ECM.

## 3. Discussion

Pro-oxidant therapies for cancer, such as CAP, have the potential to eliminate cancerous cells by exceeding their antioxidant capacity and by sensitizing cancer cells to chemo- and radiotherapy. Previous studies have shown the ability of CAP to eliminate PDAC cells in vitro, in ovo, and in vivo. However, the effect of CAP on stromal cells present in the TME and responsible for supporting the progression of cancer is still unknown. Here, we used a vascularized in ovo CAM model to generate single and co-cultured tissue of RLT-PSC and Mia PaCa-2 cells to study the effect of CAP on PSCs. The use of this xenograft model provides a more realistic setting for the development of solid PDAC tumours, which includes other cells of the TME. Our findings demonstrate that CAP reduces the growth of PDAC tissue in ovo, without significantly affecting the expression of activation markers and ECM remodelling factors in PSCs.

The response of cancer cells to oxidative stress is impaired when the multiple subcellular compartments are affected simultaneously, which can lead to cell death via multiple mechanisms. In this study, we determined that CAP treatment of co-cultured RLT-PSC and Mia PaCa-2 in ovo reduced the tissue weight. This correlates with the reduction of the number of Mia PaCa-2 cells present in the co-cultured tissue, which suggest that the reduction in weight is due to the elimination of Mia PaCa-2 cells. Although the reduction in the number of CD44^+^ Mia PaCa-2 cells could be due to a decrease in the expression of this marker, we have shown in a previous study that CAP increased the expression of CD44 in U87-MG and A375 cells [22]. It is likely that our findings correspond to the elimination of Mia PaCa-2 cells, rather than to the reduction of CD44 expression in PDAC cells. Our results are in agreement with previous findings with in vitro co-cultures of RLT-PSC and Mia PaCa-2 three-dimensional spheroids, which decreased in size after CAP treatment [27]. This was also observed in in ovo studies, where CAP evoked apoptosis in Mia PaCa-2 tumours treated with the kINPen [27,28].

Current efforts are focused on developing therapies that could overcome the desmoplastic stroma to eliminate PDAC cells. However, inhibiting key pathways in stromal cells [39,40] or simply eliminating the carcinoma-associated fibroblasts [41] can lead to more aggressive forms of PDAC due to the elimination of stromal components required for normal tissue homeostasis [42]. Considering the importance of PSCs in PDAC stroma, we have assessed the effect of CAP in both PSCs and PDAC cells. Interestingly, RLT-PSCs in single or co-cultured tissue were not negatively affected by CAP. It is worth mentioning that the active state of RLT-PSCs was unaffected, as the protein and mRNA expression of ACTA-2, GFAP, and vimentin remained unchanged. It is possible that the variation in MMP-1 expression observed in RLT-PSC tissue (fold change > 3; *p* = 0.061) reflects a transient effect in the samples treated with CAP. We set the cut-off value based on two conditions: fold change > 2, to assess the biological relevance, and *p* < 0.05, to assess the statistical significance [43]. These wo criteria combined are commonly used to identify more biologically meaningful changes in gene expression than only one of them alone [44]. In the future, MMP-1 mRNA levels could be tested in a larger sample size to determine if the variation observed in this study is of genuine biological interest. CAP did not affect the mRNA expression of GFAP in co-cultured tissue (24 h p.t.), and there was no variation in the MFI of GFAP in CAP-treated co-cultured tissue. This could suggest that CAP does not affect GFAP expression in RLT-PSC cells. However, we observed an increase in the number of cells expressing the protein. This transcript/protein discordance could be due to regulatory processes that control gene and protein expression. It is possible that CAP-treated samples experienced transient shifts in mRNA concentrations that returned to baseline values after some time, while proteins, once switched to the new steady state, remained stable due to the regulation at the protein level [45]. However, this should be further explored using statistical tools to dissect the regulation of both gene and protein expression. While previous studies have reported that CAP-treated solutions have a cytotoxic effect in PSCs in vitro [30,31], the direct application of CAP to in ovo tissue did not alter the tissue growth or proliferative state of RLT-PSCs. These findings are important, as the elimination of stromal cells in PDAC has been linked to reduced survival of transgenic mice and patients [41]. Additionally, RLT-PSCs have an activated profile [35], and the reactive species generated by CAP did not reverse or increase the expression of the activation markers. Based on our findings, it is unlikely that CAP could normalize the function and behaviour of PSCs, but it is possible that it does not boost their activity. Further studies to determine the secretion of key signalling factors involved in the crosstalk between PDAC cells and PSCs upon CAP treatment could bring light to this issue. In addition, combination therapies of CAP to eliminate PDAC cells and to normalize the activity of PSCs could be studied, considering the fine balance required to prevent tumour progression.

We have focused this study on the effect of CAP in PSCs because the stroma regulates the molecular interactions with the ECM, is responsible for the remodelling of the TME in PDAC, and facilitates the invasion and metastasis of cancer cells [46]. The matrix metalloproteinases (MMPs), mostly secreted by PSCs, but also by PDAC cells, participate actively in this process [47]. The gelatinases MMP2 and MMP9, together with other markers such as vimentin, are considered biomarkers of EMT in PDAC [48,49]. In this study, we found that CAP treatment of co-cultured tissue did not affect the expression of MMP2 and MMP9 in RLT-PSC cells, however, there was an increase in the percentage of Mia PaCa-2 cells expressing MMP2 and MMP9. This was not accompanied by an increase in vimentin expression for samples measured 72 h after CAP treatment. Several studies have shown that MMP2 and MMP9 expression is upregulated in PDAC patients [50]. The pro-angiogenic and tumour-growth-promoting role of MMP9 is well established and it has been correlated with poor survival [51]. However, the depletion of MMP9 make the tumour cells more invasive and metastatic, a process that is facilitated by the presence of CD11b^+^ inflammatory monocytes expressing pro-invasive cathepsin B and the secretion of interleukin-6 [52,53]. It has been reported that CAP can reduce the migration and invasion of HeLa cells by downregulating MMP9 expression [54]. In addition, the application of CAP-treated solutions to ovarian cancer cells in vitro has been shown to decrease MMP9 but not MMP2 mRNA expression [55]. A previous report demonstrated that CAP did not induce changes in the expression of vimentin in the pancreatic cancer cell lines PaTuS and PaTuT in vitro, whereas in three-dimensional spheroids of Mia PaCa-2 and RLT-PSC co-cultures, CAP did not enhance the migration of either cell population outside the spheroids [27]. Previous studies on breast cancer cell lines have suggested that direct and indirect application of CAP could inhibit EMT, being more effective against those with a mesenchymal phenotype [56,57]. In addition, the combination of CAP with silymarin nano emulsion on melanoma cells decreased the expression of EMT markers while reducing the tumour weight and size [58]. CAP treatment on the PDAC cell lines PaTuS and PaTuT in vitro demonstrated that the EMT marker ZEB1 was slightly increased 24 h p.t. (*p* > 0.05) [27]. However, it is worth considering that the conversion of a cell to full EMT requires time, and longer periods of monitoring to identify significant changes in gene and protein expression might be necessary [59]. Moreover, due to the dynamic and plastic nature of the EMT process, it should be considered that cells in a hybrid EMT state (not fully transitioned to a mesenchymal phenotype) could revert to the epithelial phenotype [60]. In this study we used the in ovo model to generate single and co-cultured tissue to assess the effect of CAP on PSCs. For this purpose, our samples were collected at a maximum of three days post treatment due to ethical reasons. Although it is possible that the CAP treatment used here could induce EMT in Mia PaCa-2 cells that survived the treatment, further studies to fully determine the EMT transition state of PDAC cells after treatment are needed.

The complex TME involves not only PSCs and PDAC cells, but also endothelial, nervous, and immune cells, which altogether determine the treatment outcome. Pro-oxidant therapies such as CAP that could help controlling and eliminating cancer cells need further study to determine the safety of the treatment on other TME components. While we acknowledge the limitations of our study in our 3D model, our findings provide evidence that can be further expanded to fully disclose the effect of CAP on more complex tumours in the presence of other cells of the TME.

## 4. Materials and Methods

### 4.1. Cell Lines and Reagents

The human pancreatic cancer cell line Mia PaCa-2 was obtained from the Cell Line Service GmbH. Cells were grown in Dulbecco’s modified Eagle’s medium (DMEM) supplemented with 10% foetal bovine serum (FBS, Gibco, Fisher Scientific, Merelbeke, Belgium), 2 mM L-glutamine (Life Technologies, Eggenstein, Germany), 100 U/mL penicillin, and 100 μg/mL streptomycin (Gibco, Fisher Scientific, Merelbeke, Belgium). The human pancreatic stellate cell line RLT-PSC (developed at the Faculty of Medicine of the University of Mannheim, kindly provided by Prof. Ralf Jesenofsky) was cultured in DMEM-F12 medium (Gibco, Dreieich, Germany) and supplemented with 10% FBS, 2 mM L-glutamine, 100 U/mL penicillin, and 100 μg/mL streptomycin. Cell cultures were maintained at 37 °C and 5% CO_2_. Cells were transduced with the Nuclight Red or Green Lentivirus reagents (Essen Biosciences, Ann Arbor, MI, USA) for monitoring.

### 4.2. Chicken Chorioallantoic Membrane Assay (CAM Assay)

In ovo experiments were performed as previously described [23]. Briefly, 4-day-old, fertilized chicken eggs were incubated in a horizontal position and constant turning for 1 day at 37.7 °C and 65% humidity in an egg incubator (Ova-Easy 100, Brinsea, Veenendaal, The Netherlands). On day 5, the upper pole was pierced to allow the repositioning of the air sac. On day 7, 2 × 10^6^ cells per egg were mixed with 15 µL growth reduced factor Matrigel (8.6 mg/mL; Corning, Amsterdam, The Netherlands) and implanted inside a sterile silicone ring placed onto the CAM. The tissues were CAP-treated on day 11 and collected 24 h and 72 h post treatment. After being weighed in a precision balance (Mettler Toledo, Fisher, Merelbeke, Belgium), the tissues were then processed for downstream assays accordingly.

### 4.3. kINPen IND Plasma Jet

The kINPen^®^ IND plasma device (neoplas tools, Greifswald, Germany) was used as described before [28]. Plasma treatments were done using high-purity Argon (99.999%, Air Liquide, Herenthout, Belgium) at 2 standard litres per minute. Tissues were treated for 60 s, maintaining an approximate distance of 10 mm between nozzle and tissue (the tip of the plasma plume touching the surface of the tissue).

### 4.4. Immunofluorescence Assays (IF)

Tissue was fixed with 4% paraformaldehyde and paraffin embedded. Sections of 5 µm were cut, deparaffinised and rehydrated prior to staining. Antigen retrieval was done with citrate buffer (10 mM, pH 6) for 20 min at 96 °C for vimentin, GFAP, and costaining of CD44 and MMP2 and MMP9. For costaining of α-smooth muscle actin (ACTA2) and MMP2 or MMP9, antigen retrieval was done with buffer pH 9 for 20 min at 96 °C. After cooling down for 30 min at room temperature (RT), the microscope slides were washed twice in tris buffer saline with 0.3% Triton X-100 (TBST) for 5 min. Slides were blocked in TBST with 10% bovine serum albumin (BSA, A9418, Sigma Aldrich, Saint Louis, MO, USA), goat serum (ab7481, Abcam, Cambridge, UK), or donkey serum (ab7475, Abcam) (according to the origin of the secondary antibody) for 2 h at RT. Slides were incubated overnight at 4 °C with the primary antibodies diluted in TBST supplemented with 1% of the corresponding serum. Slides were washed twice with TBST and incubated with the secondary antibodies for 1 h at RT. Slides were chemically bleached with Sudan Black B in 70% ethanol (SBB) for 20–30 min to reduce autofluorescence, as described before [61]. Afterwards, the slides were washed trice and mounted with VECTASHIELD HardSet antifade mounting medium with DAPI (Vectorlabs, Burlingame, CA, USA). Primary antibodies used: GFAP (HPA056030, Atlas Antibodies, 1/200, Bromma, Switzerland); ACTA-2 (M0851, Dako, 1/200); Vimentin (Abcam ab92547, 1/500, Cambridge, UK); CD44 (3570S, Cell Signaling Technologies, 1/400, Danvers, MA, USA); MMP2 (40994, Cell Signaling Technologies, 1/400, Danvers, MA, USA); MMP9 (13667, Cell Signaling Technologies, 1/100, Danvers, MA, USA). Secondary antibodies used: Donkey Anti-Mouse IgG H&L, Highly Cross-Adsorbed, Alexa Fluor 594 (A-21203, Thermo Fisher, Waltham, MA, USA); Goat Anti-Rabbit IgG H&L, Alexa Fluor 488 (ab150077, Abcam, Cambridge, UK). Sections were imaged with a Zeiss AxioImager Z1 microscope (Carl Zeiss, Jena, Germany) equipped with an AxioCam MR v3.0.

### 4.5. Immunohistochemistry (IHC) Assays

For Ki67, sections of 5 µm were cut, deparaffinized, and rehydrated prior to staining. Antigen retrieval was done with citrate buffer (10 mM, pH 6), at 96 °C for 20 min. Sections were permeabilised in 0.1% Tween-20 and blocked with 3% H_2_O_2_ in PBS (10 min, RT). Slides were incubated with the primary antibody for 30–40 min at RT (1/75 dilution; Mouse Anti-Human Ki-67 Antigen, Clone MIB-1, Agilent, Santa Clara, CA, USA), followed by incubation with the secondary antibody (30 min at RT; Envision Flex HRP). We used diaminobenzidine to visualize the positive staining and haematoxylin to counterstain. Sections were imaged with a Leica ICC50 E microscope using the Leica Application Suite EZ v3.4.0.

### 4.6. IHC Image Processing

Ki67 immunohistochemistry scoring was done using QuPath (open-source software [62]). Positive cell detection was used with the following settings: detection image, optical density sum; requested pixel size, 0.5 µm; background radius, 8 µm; median filter radius, 0 µm; sigma, 1.5 µm; minimum cell area, 10 µm^2^; maximum cell area, 400 µm^2^; threshold, 0.1; maximum background; intensity, 2. The intensity threshold parameters were set as follows: 1+ = 0.2; 2+ = 0.4; 3+ = 0.6.

### 4.7. IF Image Processing

**Figure A1:**
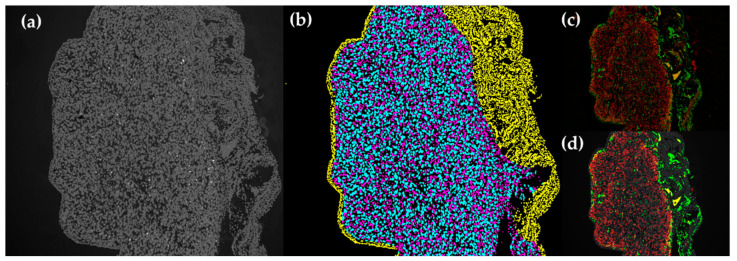
Classification of tissue categories in IF images. (**a**) Original DAPI image. (**b**) Cell labelling: cyan = human cells, yellow = chicken cells in the periphery of human tissue (CAM), magenta = chicken cells in the human tissue. (**c**) Original fluorescent images. (**d**) Segmented fluorescent images.

IF images of 10× magnification were processed using a script in Python to take the mean fluorescent intensity and fluorescence positive counts in each tissue slide. This was done after individual cells in each tissue had been classified into one of the three categories: (1) human cells, (2) chicken cells in the periphery of human tissue (corresponding to the CAM), or (3) chicken cells in the human tissue (Figure A1).

#### 4.7.1. Nuclear DAPI Segmentation

First, the DAPI image was segmented in order to produce a binary mask of individual nuclei. The raw DAPI channel was normalized from UINT8(0-255) to float(0-1) range. Background noise was removed using a minimum filter of 50 pixels and the image was saturated to the 5 and 95 percentiles. Afterward, all values above a threshold of 0.4 were considered true. An area filter to remove objects above a size of 10 pixels and below 100 pixels was applied. Lastly, nuclear objects with an area above 200 pixels were passed into a routine which would split them. Touching nuclei were separated with a watershed transform using local maxima of the DAPI signal and the distance transform of the mask. This step is to ensure proper classification, as nuclear size is an essential parameter to the delineation of chicken cells. This method is derived from the image process and analysis steps as detailed in [63,64].

#### 4.7.2. Tissue Classification

Second, features for each nucleus were collected and used for classification. These features include the nuclear area, eccentricity, and average distance from each nucleus to the next 3 nuclei (k-3 density). The values were fed into a pretrained linear classifier, and each nucleus is assigned a class. Membranous structures at the edge of the section, corresponding to the CAM tissue, were labelled as chicken tissue and filtered out of the analysis for human cells. The classifier was pretrained using a set of curated training images selected from the dataset, which were selected to include a wide representation of the CAM tissue structure. The process for this is derived from the hand engineered feature method detailed in [65].

#### 4.7.3. Fluorescent Signal Measurement

Finally, using the labelled nuclear regions, the fluorescent signal was measured. First, the pixels surrounding each of the nuclei were indexed to each of the nuclei in order to create a labelled masking of the cytoplasm of each cell. The mean fluorescent signal was measured for each of the labelled cell regions (nuclear overlapping, cytoplasmic overlapping) for each cell then averaged together for each slide.

Fluorescent images were filtered similar to the DAPI image using a minimum filter to quantify and remove background noise. Fluorescent images were then threshed at 0.4. Fluorescent images were not saturated before segmentation as were the DAPI images. Counts were determined by using the nuclear cell mask to index each of the fluorescent positive images. If the region intersecting where a cell was detected and the fluorescent signal was positive, and if that region was larger than 5 pixels, then that cell was considered to be positive for that signal.

#### 4.7.4. Quantification of ACTA-2^+^ Cells

The masks for certain fluorescent stains were further filtered for that stain to eliminate areas that do not morphologically correspond to stellate cells. For tissue stained for ACTA-2, masked objects must be larger than 20 pixels and have an elongated morphology, defined as having a perimeter to area ratio higher than 0.5. This is to ensure the regions that are considered positive for ACTA-2 are characteristic to stellate cells. By using this method, the presented metrics consider only the signal from stellate cells present in the tissue, excluding any chicken cells infiltrated into the tissue or in the surroundings.

### 4.8. Tissue Homogenization and RNA Extraction

Excised tissue was collected into RNAlater and stored at −20 °C until processing. Samples were homogenized using GentleMACS M tubes (Miltenyi Biotec, Bergisch Gladbach, Germany) and the RNA was extracted using an Rneasy Plus mini kit (74134, Qiagen, Hilden, Germany) following the manufacturer’s instructions. RNA was eluted in 50 µL Rnase-free water and quantified using a NanoDrop Spectrophotometer (Thermo Fisher Scientific, Waltham, MA, USA). RNA quality was determined using a DNF471-SS RNA kit (Agilent, Santa Clara, CA, USA) and a Fragment Analyzer Automated CE system (Advanced Analytical Technologies, Ankeny, IA, USA). Results were visualized using PROSize 3.0 data analysis software (Agilent, Santa Clara, CA, USA). The RNA concentration was standardized to 10 µg/100 µL.

### 4.9. Reverse Transcription Quantitative PCR (RT-qPCR)

The RNA was amplified using a Power SYBR RNA-to-CT 1-Step Kit (4389986, Thermo Fisher Scientific, Waltham, MA, USA). The quantitative polymerase chain reactions were done with a CFX384 Touch Real-Time PCR Detection System (Bio-Rad, Hercules, CA, USA) and analyzed with Bio-Rad CFX Maestro 1.1 software, v4.1.2433.2019. PCR of each sample was done in triplicate for the target gene and the 2 housekeeping genes. The list of primers can be found in Appendix A, Table A1.

### 4.10. Statistical Analysis

One-way ANOVA followed by Tukey’s multiple comparison test was performed using Prism v9.3.1 (GraphPad Software, San Diego, CA, USA). Statistical significance was set at *p* ≤ 0.05.

## 5. Conclusions

Our findings support the ability of CAP to eliminate Mia PaCa-2 cells, without altering the growth and phenotype of RLT-PSC cells, in the vascularized in ovo model. This is an important finding, as the elimination of PSCs from PDAC tissue is detrimental. However, Mia PaCa-2 cells from co-cultured tissue presented an increase in MMP2 and MMP9 72 h after CAP treatment. This should be considered in future studies to determine the effect of CAP on EMT in Mia PaCa-2 cells.

## Data Availability

The data presented in this study are available on request from the corresponding author.

## Appendix A

**Table A1:** List of primers used for quantitative real-time PCR.

Gene	Forward Primer (5′ to 3′)	Reverse Primer (5′ to 3′)
ACTA-2	CTAAGACGGGAATCCTGTGAAG	ATGGATGGGAAAACAGCCCT
GFAP	TGCCTATAGACAGGAAGCAGATG	TCCTCCTCCAGCGACTCAAT
vimentin	GGCTCGTCACCTTCGTGAAT	GAGAAATCCTGCTCTCCTCGC
e-cadherin	GGGCTGGACCGAGAGAGTTT	GTTAGCCTCGTTCTCAGGCA
n-cadherin	CGAAGGATGTGCATGAAGGAC	TGCAGTTGCTAAACTTCACATTG
SNAI2	TTGTGTTTGCAAGATCTGCGG	TTCTCCCCCGTGTGAGTTCTAA
MMP1	CAGGGGAGATCATCGGGACAA	GGCCTGGTTGAAAAGCATGAG
TIMP2	TATCTACACGGCCCCCTCCT	CGGCCTTTCCTGCAATGAGATA
fibronectin 1	AACAAACACTAATGTTAATTGCCCA	TCGGGAATCTTCTCTGTCAGC
B2M	TGCTGTCTCCATGTTTGATGTATCT	TCTCTGCTCCCCACCTCTAAGT
PMM1	GCTTCGACACCATCCACTTCTTTG	AGATCTCAAAGTCGTTCCCACCAG
